# Dynamics of Spin Crossover Molecular Complexes

**DOI:** 10.3390/nano12101742

**Published:** 2022-05-19

**Authors:** Thilini K. Ekanayaka, Krishna Prasad Maity, Bernard Doudin, Peter A. Dowben

**Affiliations:** 1 Department of Physics and Astronomy, University of Nebraska, Lincoln, NE 68588, USA; thiliniek@huskers.unl.edu; 2 Institut de Physique et Chimie des Matériaux de Strasbourg, Université de Strasbourg CNRS, UMR 7504, 23 Rue du Loess, BP 43, CEDEX 2, 67034 Strasbourg, France; krishna.maity@ipcms.unistra.fr

**Keywords:** spin crossover molecules, intermediate excited states, spin state switching speed, time domain laser spectroscopy, pump and probe

## Abstract

We review the current understanding of the time scale and mechanisms associated with the change in spin state in transition metal-based spin crossover (SCO) molecular complexes. Most time resolved experiments, performed by optical techniques, rely on the intrinsic light-induced switching properties of this class of materials. The optically driven spin state transition can be mediated by a rich interplay of complexities including intermediate states in the spin state transition process, as well as intermolecular interactions, temperature, and strain. We emphasize here that the size reduction down to the nanoscale is essential for designing SCO systems that switch quickly as well as possibly retaining the memory of the light-driven state. We argue that SCO nano-sized systems are the key to device applications where the “write” speed is an important criterion.

## 1. Introduction

Transition metal-based spin crossover (SCO) molecular complexes have attracted much interest due to their remarkable bistable properties and potential applications [1,2,3,4], in particular for molecular memory devices [5,6,7,8]. A better understanding of the mechanisms responsible for their change of spin state is motivated by the ultimate goal of investigating charge transfer through a single molecule junction [9,10,11,12,13]. Mastering the bistable properties of molecular SCO systems is of paramount importance to realizing a significant advance in molecular electronics such as molecular switches [13,14,15], molecular rectifiers [16,17,18,19,20,21,22], sensors [23,24], and molecular transistors [13,25,26]. Applications beyond data storage devices can then be envisioned, in particular for photon conversion or charge transfer in solar cells [27,28,29].

In the context of device applications, the speed of the transition between the molecular magnetic states is a key parameter for a figure of merit [8]. In characterizing switching of the molecular spin state, the intrinsic switching speed of spin crossover complexes is of paramount importance to establishing the limits to the write delay time in molecular devices. Even though the often limited electronic state switching speed is considered a major limitation of molecular electronics devices, recent studies showed integrated molecular rectifiers with ultrathin molecular junctions are able to operate at a 10 MHz frequency [21], and even molecular diodes capable of operation up to 17 GHz [19]. Identifying the spin state switching mechanisms and the possible different routes to spin state switching are essential to understanding the intrinsic limits to the switching speed. It must be acknowledged that different spin state switching pathways may have different intrinsic time scales. Moreover, the dynamics of the excited states in the spin state conversion process might be better controlled by having a clear understanding of the excited state potential surfaces. SCO switching speed measurements that lead to an improved understanding are important to identifying suitable molecular materials and materials combinations or devices design optimization and thus of considerable interest.

**Figure 1:**
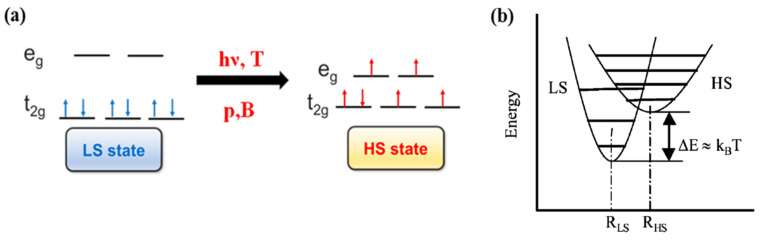
(**a**) The electronic configuration of low spin (LS) and high spin (HS). (**b**) The relative potential energy diagram of low spin (LS) and high spin (HS) states. Reprinted with permission from Ref. [40]. Copyright 2004 Springer.

In transition metal molecular complexes, the spin crossover phenomenon is a consequence of the energy splitting of d orbitals of the central metal ion into *t*_2*g*_ and *e_g_* orbitals (Figure 1a) in the ligand field [30,31]. As a result, depending on the nature of this ligand field, a SCO molecular complex may exist either in the low spin or high spin states. The transition between the different spin states may be triggered by external stimulus such as temperature [30,32], light [33,34], pressure [32,35], magnetic pulses and electric field [1,30,32,36,37]. This spin crossover phenomenon can be observed for transition metal ions in the first transition series with d^4^ to d^7^ configurations [3,30]. The change in the electronic configuration of the molecular complex, for each electronic spin state resulting from the SCO phenomena, can drastically modify the physical properties like the molecular structure, the optical properties, the magnetic susceptibility, the volume [1,30,31,32,38,39] and conductivity [8]. For SCO molecular complexes with transition metal ions with d^6^ electronic configuration, the spin state transition is between a low spin (LS, $t_{2g}^{6}e_{g}^{0}$, ^1^A_1_) diamagnetic state (ground state) of the central metal ion to high spin (HS, $t_{2g}^{4}e_{g}^{2}$, ^5^T_2_) paramagnetic state (excited state). Figure 1b shows the potential energy of LS and HS states with respect to the metal-ligand distance. The HS state potential is associated with a larger metal-ligand distance with respect to the LS state potential and the vertical shift between the bottom of the two potential wells falls within the range of the thermal energies [31,40,41].

For spin crossover complexes, studies that explore thermal-induced and light-induced spin state transitions are common [42]. The spin state transition, driven by temperature, is due to the large entropy gain at the HS state resulting from electronic and vibrational contributions [43]. The spin state switching, induced by light, can results in an efficient trapping into a metastable spin state [44,45,46]. The light-induced excited spin-state trapping (LIESST), where a SCO complex is perturbed from the low temperature stable (usually) LS spin state into a metastable (most commonly) HS state was discovered nearly four decades ago [44,45,46,47,48]. At low sample temperatures, typically below 50 K, the LIESST effect results in an excitation to a HS state that is very long lived. At higher temperatures (usually at temperatures above 50 to 70 K), this metastable HS state, typically, very quickly relaxes back to the LS state as long as one is below the effective LS to HS spin state transition temperature, revealing a transient HS state lifetime typically in a range from nanoseconds to milliseconds, as discussed below. With the discovery of LIESST, Decurtins et al. [47,48] showed that the relaxation from HS to LS is suppressed appreciably as the system remains in HS state under continuous irradiation of light. This discovery of optical pumping to the HS state created interest in the trapping mechanism of an SCO complex in this metastable HS state and the properties and parameters that govern its lifetime. LIESST can also be observed in crystalline molecular solids, which raised the interest in studying the influence of interaction between the metal ions (cooperative effects) on the spin dynamics of SCO systems [49,50].

With the advent of increasingly shorter pulse laser systems, spin state switching, and the HS state relaxation times could be studied in much greater depth. The result is much expanded literature on the light-induced switching dynamics in spin crossover molecular complexes. Ultrafast pump probe systems in which ultra-short laser pulses have been used to pump the initial state and a secondary laser pulse is then employed to probe the final state, are ideally suited to the time domain studies. Note, however, that the practical requirements for repetitive measurements limit most studies to the studies of the transition to the metastable state, under temperature conditions that make the excited state short-lived. This approach to the study of optically driven state transitions is the method of choice to characterize the spin state switching in the diluted and bulk system over an extended time scale [51,52,53]. There are, nonetheless, a variety of experimental tools, using different spectroscopies such as X-ray spectroscopies [54,55,56,57], X-ray scattering [58], ultrafast time resolved optical spectroscopy [54,59], picosecond/femtosecond time resolved diffraction [54,60], transient X-ray absorption spectroscopy [61,62], ultrafast time resolved X-ray absorption near edge structure (XANES) [63,64], transient reflectivity [52] and Raman spectroscopy [65], which have provided insight into the structural and electronic dynamics of the SCO systems. Such efforts have provided insight into the lifetime of the excited state for SCO systems with different ligands and identifying what parameters affect the excited state time stability [30].

**Figure 2:**
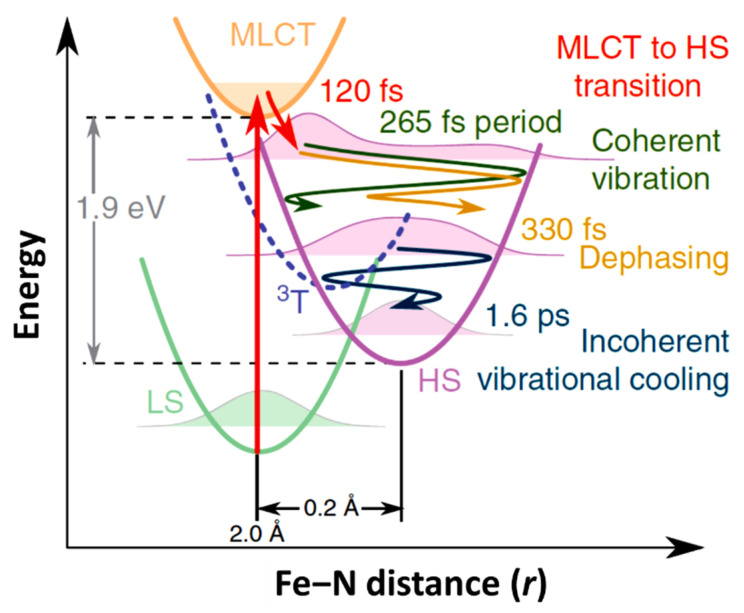
The schematic representation of structural dynamics of [Fe^II^(bpy)_3_]^2+^ in solution during the light induced spin state conversion with respect to the Fe-N distance reaction coordinate. The decay of the metal-ligand charge transfer (MLCT) state occurs with 120 fs with energy dissipation. The system then undergoes a coherent vibronic decay (at 265 fs) followed by a damping with a time constant of 330 fs. The vibrational cooling occurs within 1.6 ps [67].

For SCO molecules, where voltage-induced switching remains in its infancy, light-induced strategies are currently best suited for spin state switching time domain studies. Thus, this review concentrates mostly on the light induced switching dynamics from low spin (LS) to high spin (HS) for both SCO molecules in solution and solids, including thin films and nanoparticles. As shown in Figure 2, several steps characterize the spin state transition processes that apply to SCO molecules under irradiation. The switching dynamics are rather complicated as the transition path from the photo-induced excited state (the metal-ligand charge transfer or MLCT state) involves several out-of-equilibrium physico-chemical processes, as shown in Figure 2. In this review, after explaining the different energy levels involved in the LS → HS transition, we then review the evolution of the experimental picture from the single molecule to the solid before addressing scaling. As spin crossover molecules in solution can be considered as independent molecules without substantive cooperative effects, their switching properties provide information on the single molecule switching time scale, in the ps range (Figure 2). The switching dynamics of SCO materials in solid form are mediated by interacting SCO molecules as the intermolecular cooperative behavior is important. Photoswitching dynamics in solids follow a complex trajectory in which different processes and different length and time scales are involved. The pertinent length scales ranging from molecular to macroscopic. These processes exhibit dynamics from the sub-picosecond (molecular) time scale to the microsecond thermal equilibrium restoration time scale [51,66]. We, therefore, include in this review a discussion of the intertwined size and time scales in these SCO systems as thus far ascertained from experiment. Finally, the reversibility of the transition, or more specifically the dynamics of HS → LS photo-induced switching, while much less documented experimentally, will also be discussed here.

Our aim here is to emphasize the importance of size reduction to increase the speed of the transition. Applications require mastery of cooperative effects, allowing us to take advantage of recent results making possible the identification of the appropriate SCO system size range for device applications, making spin crossover molecular nanomaterials highly unique and attractive.

## 2. Light Induced Spin Crossover Elementary Processes

**Figure 3:**
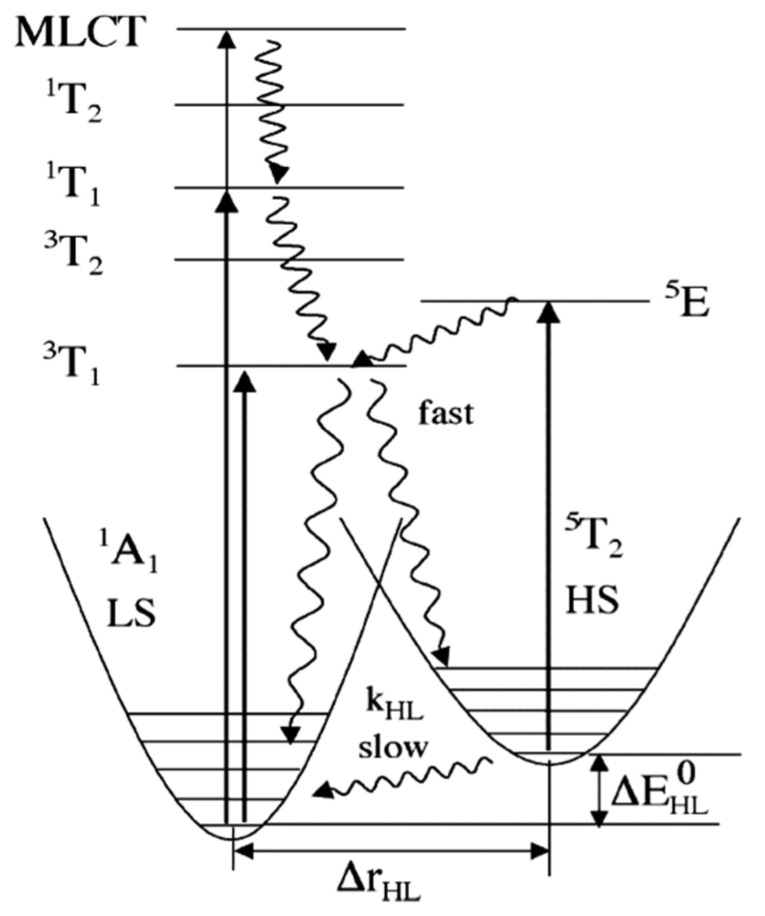
The energy diagram includes the potential energy surfaces of all the energy states involved in LIEEST and reverse LIEEST (indicated by curly arrow) [70,71,72]. Reprinted with permission from Ref. [72]. Copyright 2004 Springer.

The light induced spin state switching of a Fe (II) based molecular complex, between the electronic LS singlet state and the HS quintet state, happens when the photon energy is at resonance with the allowed metal ligand charge transfer states (MLCT) or d-d absorption band. Direct excitation from low spin to the high spin is not allowed by the spin selection rule (i.e., ΔS = 2 is not optically allowed) [47,49,68,69]. Therefore, the excitation to the various high spin states must happen via lower-lying ligand field (LF) ^1,3^T states or via the singlet metal ligand charge transfer states ^1^MLCT. Here, the superscript notation 1 and 3 in MLCT and ligand field states indicate the spin multiplicity of the states. The potential energy surfaces of all the energy states based on the optical absorption studies are shown in Figure 3. The light-induced spin state transition from the LS to HS states, based on the LIESST effect, should involve a decay cascade with at least two intersystem crossings of ΔS = 1. The transition can also occur through an intersystem crossing between adjacent molecules, in a non-radiative process that occurs between two electronic states with different spin multiplicities, like an excited singlet state to a lower lying triplet state. The initial optical absorption, at around 514 nm, will either initially excite from the LS state to the singlet metal ligand charge transfer state, ^1^MLCT state, or the ^1^T_1_ LF state [47,49,70,71]. Then the first intersystem crossing (ISC) with ΔS = 1 will occur either from the singlet metal ligand charge transfer state, ^1^MLCT, or from the ^1^T_1_ state to ^3^T_1_. The second ISC step with ΔS = 1 will be the transition from the ^3^T_1_ state to the high spin state, ^5^T_2_. The HS state configuration, (*t*_2*g*_)^4^(*e_g_*)^2^ as schematically shown in Figure 1, corresponds to a metastable “ground” state ^5^T_2_. Hereafter the HS state will be referred as ^5^T_2_. At low temperatures (typically below 50 K, as noted above) where the vibronic decay mechanisms are suppressed, the spin state of the SCO complex will be trapped in the HS state. This long-lived metastable state ^5^T_2_ can relax to the LS state ^1^A_1_ by increasing the temperature, where the true ground state ^1^A_1_ LS configuration is (*t*_2*g*_)^6^(*e_g_*)^0^. The LS stated can also be retrieved by irradiating at 820 nm, inducing a transition from ^5^T_2_ to ^5^E (^5^E is a ligand field state with (*t*_2*g*_)^3^(*e_g_*)^3^ electronic configuration) through a process called reverse LIESST. It has been shown that even after prolonged irradiation at 820 nm, the resulting fraction of low spin state occupancy ($\gamma_{LS}$) is around 85%, which is due to the overlap between the transition of ^5^T_2_ to ^5^E and the transition band from ^1^A_1_ to ^5^T_2_ via spin-forbidden, short lived intermediate triplet states ^3^T_1_ or ^3^T_2_. A transition from ^1^A_1_ to ^5^T_2_ through the triplet intermediate state can also be seen under a 980 nm irradiation [70,71,72].

## 3. Dynamics of Spin State Switching of SCO Molecules in Solution

As indicated at the outset, insights into the kinetics of light-induced spin state switching of molecules in solution were first obtained by conventional ultrafast laser spectroscopy studies. The initial excited state, the singlet metal ligand charge transfer state, ^1^MLCT, populates via a Franck–Condon process, i.e., vertical transition between potential energy surfaces, and ultimately leads to the population of the lowest lying quintet state and the depopulation of the singlet metal ligand charge transfer state. Going from singlet metal ligand charge transfer state, ^1^MLCT, to the ^5^T_2_ HS state, the initial population of the metal ligand charge transfer state (^1^MLCT), in most of the SCO complexes, occurs within ≤1 ps [72], i.e., the intersystem crossings occur at the sub-picosecond time scale. Detailed observation of these state transitions was initially not possible [31,72,73,74,75,76], due to the insufficient time resolution, but this situation has evolved for the better so much more is now known.

Further studies were performed by means of pump-probe techniques, where an ultrashort laser pulse with high intensity is used as the pump for the SCO complex via an electronic excitation of the system, while the probe pulse (laser pulse, X-ray or electron) can be used to monitor the change of the excited states structure in both temporal and spectral domain. One example is Fe(II)-tris(bipyridine) ([Fe^II^(bpy)_3_]^2+^). Relaxation dynamics of aqueous Fe(II)-tris(bipyridine) ([Fe^II^(bpy)_3_]^2+^) has been characterized by Gawelda and coworkers [77,78] using femtosecond fluorescence up-conversion and transient absorption (TA) spectroscopies. After the initial optical excitation, the resulting relaxation, Fe^II^-tris(bipyridine) ([Fe^II^(bpy)_3_]^2+^) appeared to have an intermediate state, a triplet metal ligand charge transfer state ^3^MLCT, involved in the relaxation path of the singlet metal ligand charge transfer state ^1^MLCT to high spin state [77]. The triplet metal ligand charge transfer state ^3^MLCT populates ˂ 20 fs after the optical excitation. After the intersystem crossing from the singlet metal ligand charge transfer state ^1^MLCT to the triplet metal ligand charge transfer state ^3^MLCT, the decay of the triplet metal ligand charge transfer state ^3^MLCT states occurs within 120 fs [77].

**Figure 4:**
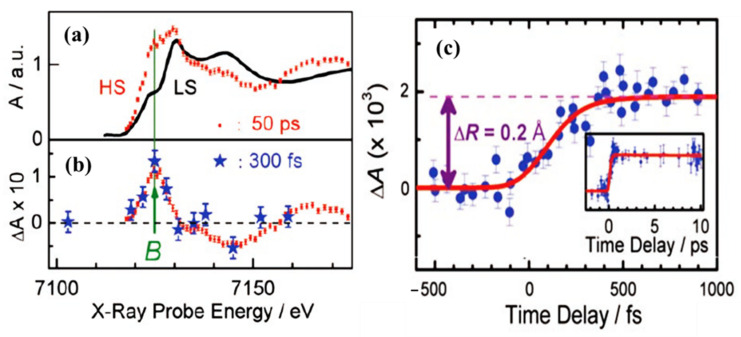
(**a**) The Fe K-edge XANES spectrum of [Fe^II^(bpy)_3_] ^2+^ high spin state (red dots) and low spin state (black curve). The HS state was extracted from the transient spectrum (**b**) and the low spin spectrum. (**b**) The transient XANES spectrum recorded at 50 ps (red dots) and at 300 fs (blue stars) after the laser excitation. (**c**) Scans of the time evolution of the B-feature signal, with respect to the laser pump/X-ray probe time delay after excitation at 400 nm. The red curve is the simulated signal assuming four-step kinetic model. The inset shows the time scan done up to a 10 ps time delay. Reprinted with permission from Ref. [79]. Copyright 2009 American Association for the Advancement of Science.

Femtosecond XANES studies showed that the population of triplet metal ligand charge transfer state ^3^MLCT relaxes to the high spin state ^5^T_2_, directly, bypassing the ^1,3^T intermediate states [79], as indicated in Figure 3. The pump and probe K-edge XANES spectra obtained for LS state (black solid line) and for HS state obtained after 50 ps delay (red dots) is shown in Figure 4a and are indicative of an increasing HS state population and Fe-N bond distortion. The transient XANES spectra recorded at 50 ps time delay and 300 fs time delay are shown in Figure 4b. There is an increment in the absorption intensity at around 7126 eV in Figure 4a which is called the B-feature [79]. This multiple scattering B-feature is attributed to Fe-N bond elongation associated with LS to HS spin state transition. Elongation of the metal to ligand bond length (Fe-N) is an easily identified structural change that occurs with the spin transition in Fe(II) SCO complexes [46,80]. The average change in the Fe-N bond length associated with the spin transition phenomena is about 0.2 Å with respect to the ground state. This structural signature of the spin state transition means that time resolved X-ray scattering can be used to probe the spin crossover dynamics.

Other SCO systems like [Fe^II^(tren(py)_3_)]^2+^ [81] and [Fe(terpy)_2_]^2+^ [82] also show similar Fe-N bond length elongation upon spin transition. Figure 4a shows the signal at the B feature as a function of delay time, up to 1 ps. It shows a steep rise that becomes steady after 300 fs [79]. This suggests that the system is already in the HS state, as confirmed by the transient spectrum in Figure 4b in which the energy scans recorded at time delay of 50 ps agree with the spectrum recorded at a time delay of 300 fs. The calculated simulation of the signal implies that the time taken to reach the HS state corresponds to the decay time of the triplet metal ligand charge transfer state ^3^MLCT. This tends to confirm that the hypothesis of a spin conversion cascade occurs via the ^3^MLCT, bypassing ^1,3^T intermediate states [79].

The suggestion that there was a direct intersystem crossing, from the initially excited MLCT state to the HS state, with a time constant of 120 fs was made by Gawelda and co-workers [77] but was later replaced by a mechanism introduced by Zhang et al. [55]. They showed that HS to LS pathway includes the metal ligand intermediate states ^1,3^T between triplet metal ligand charge transfer state, the ^3^MLCT state, and the final high spin quintet state. In other words, there is a sequential pathway relationship with two steps: the triplet metal ligand charge transfer state, ^3^MLCT to ^3^T, and the ^3^T to ^5^T_2_, with time constants of 150 fs and 70 fs respectively [55]. A theoretical study on this type of molecular spin state transition path showed that the spin orbital coupling between MLCT and ^5^T_2_ states is weak [83]. The spin orbit coupling between the triplet MLCT state, and the HS state influences the intersystem crossing. Weak spin orbit coupling makes the direct relaxation inefficient. It has also been shown that there are more intermediate energy states involved, such as the ^3^T_1_ and ^3^T_2_, therefore transition dynamics is more complex with intersystem crossing and internal conversion (IC) processes [83]. Most of the experimental studies on spin state switching were done with the temporal resolution of 100 fs to 250 fs [55,79]. Auböck et al. performed transition absorption spectroscopy measurements on the [Fe(bpy)_3_]^2+^ SCO complex with the temporal resolution of 40–60 fs and pointed out that the direct relaxation from MLCT to HS states occurs in less than 50 fs [62]. This relaxation process is mediated by the non-total symmetric modes. There is a high frequency mode associated with the C=C elongation of the bpy ligands which creates a structural distortion in the [Fe(bpy)_3_]^2+^ SCO complex, such as a distortion N-Fe-N angle which can also support the direct relaxation [62].

The relaxation process from the MLCT to HS states is accompanied and mediated by coherent vibrations which carry away excess vibrational energy. The wave packet that reaches the HS state will undergo a vibrational cooling, inside the HS state, as the energy is transferred to the environment. Consani et al. [61] first observed the vibrational coherence in the HS state of [Fe^II^(bipy)_3_]^2+^. It was shown that the vibrational coherence occurs only at the HS state energy surface and not in the ground state bleaching. The observed oscillation wave number at 130 cm^−1^ differs from the wave number of the Fe-N stretching mode in the excited state (280 cm^−1^) and was attributed to the N-Fe-N bending mode due to ultrafast elongation of Fe-N bonds [61]. The dispersion dynamics of the wave packet, in the HS state, have also been studied using transient XANES by Cammarata and coworkers [67].

**Figure 5:**
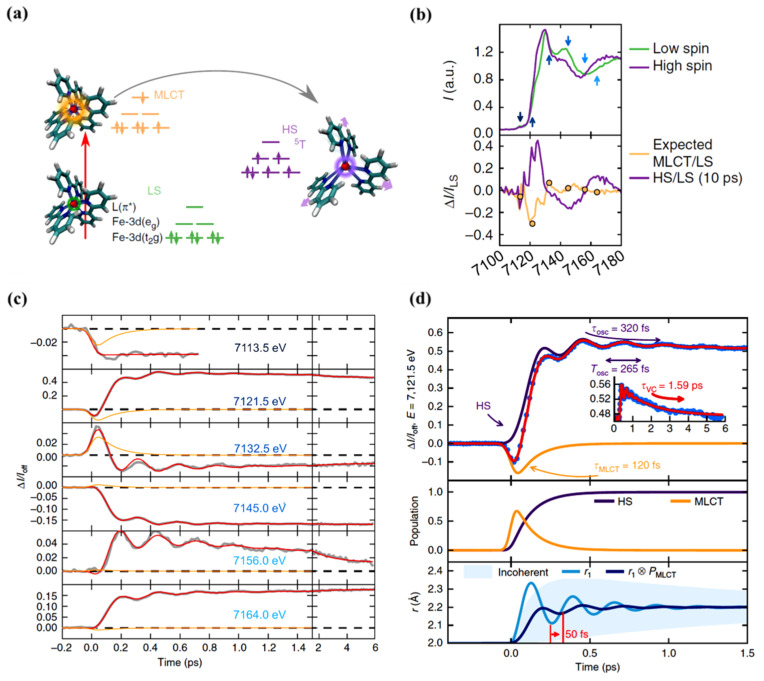
(**a**) A schematic representation of the spin state trapping by light for [Fe^II^(bpy)_3_]^2+^. The Fe atom is represented by the small red sphere bonded to N atoms of the ligand. The low spin state initially excites to the MLCT state, and then, the system relaxes to the high spin state. (**b**) (**top**) The XANES spectra of the low spin and high spin state. The arrows indicate the photon energies for which the time resolved data was taken. (**bottom**) The high spin/low spin spectra change ratio and the expected MLCT/low spin ratio. (**c**) The femtosecond transient XANES changes ΔI(t)/I_off_ after photo excitation for different photon energies. The red lines correspond to the global fit of the entire data set with an empirical model and the orange lines represent the MLCT contributions. The oscillating nature of the high spin and the nature of the MLCT state for 7121.5 eV photon energy (top panel). The population of the HS and MLCT states with time (middle panel). (**d**) The time evolution of the Fe-N distance r and average coherent oscillating trajectory, $\vec{r}\left(t\right)=r_{1}\left(t\right)\times P_{MLCT}\left(t\right)$ due to high spin population from MLCT state with respect to the time. There is a phase shift (at a delay time ~50 fs) due to an increase in the high spin population through a decrease in the MLCT population and also a reduction in the amplitude (bottom plane) [67].

As just indicated, the changes in the MLCT state occupancy with time can be characterized. The XANES spectra obtained for the LS ground state and excited state, at a time delay of 10 ps, are shown in Figure 5b (top diagram). The femtosecond transient changes to XANES, ΔI(t)/I_off_, after photoexcitation for different photon energies, is shown in Figure 5c for the region of the Fe K-edge, selected from the pre-edge region of 7113.5 eV to 7164 eV X-ray photon energies. The X-ray absorption at pre-edge region is low for the HS state compared to the LS state as the *e_g_* state becomes partly occupied and clearly shows the damped oscillations at or above the K-edge. It also indicates the relative absorption changes of the MLCT state with the time [67]. The oscillating nature of the X-ray absorption corresponding to the HS state and the nature of the MLCT state X-ray absorption, with delay time, are shown in Figure 5d (top panel) for a 7121.5 eV excitation, as an example. The lifetime of the MLCT state was found of the order of 120 fs. The middle panel in Figure 5d indicates how the population of each state changes with time, and the bottom panel depicts the time evolution of the Fe-N distance with its amplitude reduced with respect to a directly initiated damped oscillation in high spin potential [67]. An oscillation frequency of 126 cm^−1^ was found in the HS state and attributed to the activation of molecular breathing mode [67]. This molecular breathing is the in-phase stretching of all Fe-N bonds with rigid ligands. The decay of the MLCT state occurs through an intermediate state ^3^T, transitioning towards the HS state and undergoes fast damping due to energy dissipation. The system undergoes a coherent vibration with oscillation period of 265 fs (high spin breathing mode) and is damped with a time constant of 330 fs around the HS state equilibrium as the excited state system loses its energy (Figure 2). This induces a vibrational cooling inside the HS potential with a time constant of 1.6 ps [67]. Different spin crossover systems show different relaxation time constants, for example, Fe(phen)_3_](BF_4_)_2_ where (phen = 1,10-phenantroline) complex dissolved in acetonitrile has a time constant of 220 fs for the relaxation of the MLCT to the HS state and the vibrational relaxation occurs within 8.6 ps. For [Fe(tren(py)_3_)](PF_6_)_2_ dissolved in acetonitrile (where tren(py)_3_ = tris(2-pyridylmethylimino-ethyl)amine), depopulation of the MLCT and vibrational cooling of the HS state occurs within 100 fs and 10 ps respectively [67].

## 4. Dynamics of Spin State Switching in Solid SCO Molecular Systems

Two principal complications emerge when investigating ensembles of interacting SCO molecules. First, the fluence of irradiation is important. The fraction of molecules switched towards the HS state is expected to increase with fluence, and the development of their cooperative behavior can result in non-linear effects. Secondly, elastic effects cannot be neglected, as the single molecule transitions do result in changes in interatomic distances within the SCO complex leading to shape and possible volume changes. This has been extensively documented through structural studies which indicate that the two spin phases in SCO materials can be associated with differences in molecules packing [84].

**Figure 6:**
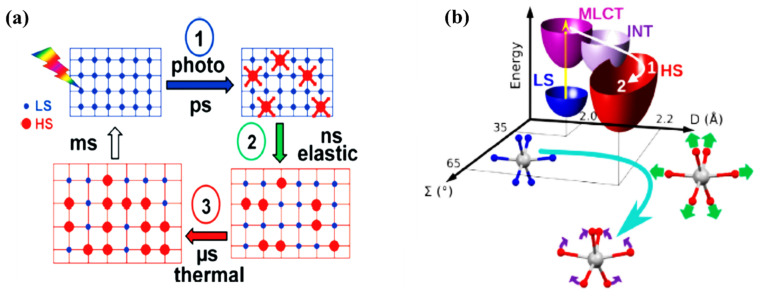
(**a**) A schematic drawing of the multiprocess switching dynamics of solid SCO materials [51]. (**b**) The schematic representation of the pathway from low spin to high spin [Fe(phen)_2_(NCS)_2_] crystal with the 2D vibronic states (D—elongation and Ʃ—bending) is indicated. The fast intersystem crossing (1), through intermediate states, drives the Fe-N elongation (2) with the generation and damping of the breathing mode followed by the activation bending mode (3). Reprinted with permission from Ref. [51] and Ref. [63]. Copyright 2009 and 2014 by the American Physical Society.

The structural dynamics of SCO solid systems, due to spin crossover transition, have (again) mostly been studied using time resolved pump-probe X-ray diffraction, X-ray absorption, and optical spectroscopies. It has been identified by Lorenc et al. [51] that the LS state to HS state switching dynamics in solid molecular SCO systems is a multistep process, which has three distinct steps, with the addition of a thermal relaxation step (Figure 6a). While this thermal equilibrium relaxation process occurs with a rather long microsecond timescale, the initial step(s) of forming the HS state molecules by photo-induced molecular switching are expected to occur on the picosecond time scale, building on understanding obtained from the study of independent molecules (Figure 6a) [51]. The photo-excited MLCT state will be depopulated via the intersystem crossing process. As just noted, the time constant of this process is different for different SCO systems and is around 200–300 fs. The photo-induced spin state switching step in solids is an ultrafast switching step which is similar to the spin-state switching observed on SCO molecules in the solutions [63]. Vibrational cooling of the excited species to the HS state can be observed in solid SCO molecular systems as well. It was observed that for a [Fe(phen)_2_(NCS)_2_] crystal, the symmetric breathing mode is first activated with a time constant of 160 fs [63]. Then the second bending mode is activated. The excitation of the second bending mode is delayed and reaches maximum amplitude at around 1 ps. Therefore, there is a sequential activation of stretching and bending modes which lead the system to oscillate in the HS state along with two reaction coordinates, namely the elongation coordinate (D) and bending coordinate (Ʃ), as shown in the Figure 6 [63]. These vibrational modes become damped as the excess energy dissipates through phonon-phonon coupling to the environment. This induces vibrational cooling in photo-switched SCO molecules in the crystal at the picosecond timescale. Even though different SCO crystals have different vibrational cooling time constants, the vibrational cooling is slower in solution. This reveals that there is better coupling efficiency of the photo-excited molecules with the lattice via the lattice phonons [63].

**Figure 7:**
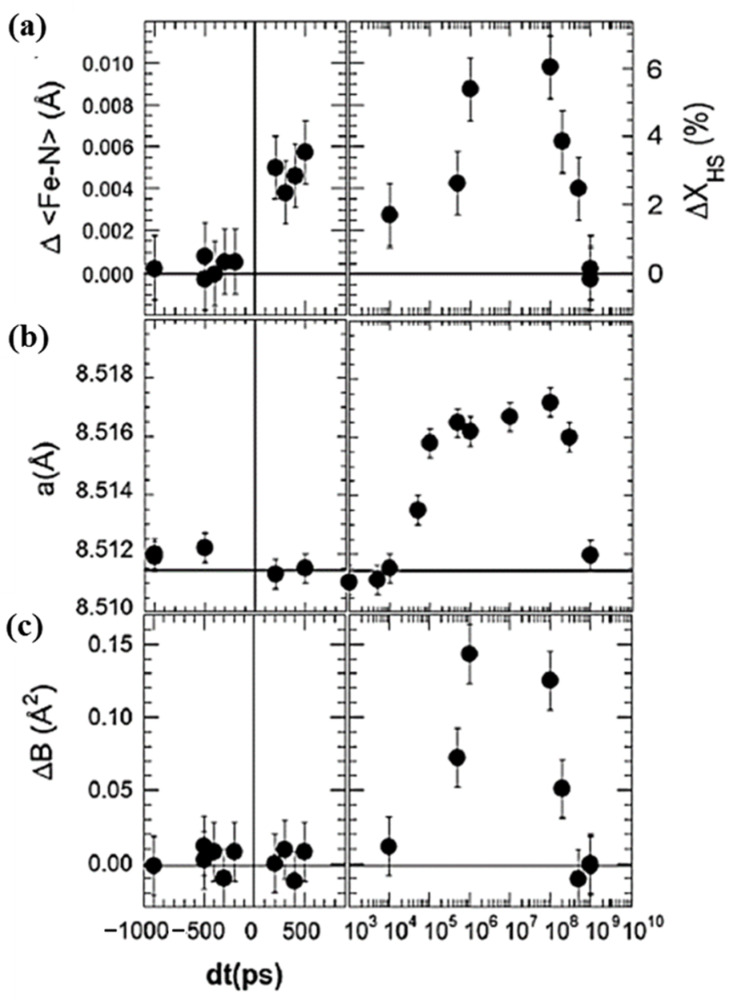
The time dependent structural change at 180 K for [(TPA)Fe(III)(TCC)]PF_6_. (**a**) The time-delay dependent average Fe–N bond length change Δ<Fe–N> and the deduced variation of ΔX_HS_. (**b**) the change in the lattice parameter <a> as a function of the time delay. (**c**) The isotropic temperature factor variation ΔB, as a function of the time delay. Reprinted with permission from Ref. [51]. Copyright 2009 by the American Physical Society.

The change in the intramolecular bond length of monoclinic [(TPA)Fe(TCC)]PF_6_ appears to accompany the change in the HS molecular fraction, with respect to time, in the initial step of the LS to HS state switching process, as shown in Figure 7a [51]. The switching of the molecules using photons is a linear process in which one photon switches one molecule. The HS fraction change is about 3%, and the change in bond distance Δ<Fe-N> ≈ 0:005 Å due to photo transformation in the first 100 ps, for the experimental conditions as shown in Figure 7a. These changes become larger at around the 100 µs time scale, at which the high spin fraction reaches around 6% and the change in bond distance Δ<Fe-N> reaches 0.01 Å [51].

The HS state fraction can also be increased by the overall sample volume expansion or pressure within the sample. There are two contributions to this volume expansion in a solid (poly-molecular condensed matter) sample. One is due to the elastic effects generated by the photo-switching of molecules from the LS to HS state, and the second one is due to the thermal lattice expansion. In Figure 7b, a delay of about 50 ns (for 100 µm laser spot size and typical sound velocity of 1000 ms^−1^) is observed before the initial photo-induced HS fraction is sufficient to result in a reorganized molecular structure that can trigger the switching of other molecules. This 50 ns time delay corresponds to the time scale required by acoustic wave propagation in a thin crystal. This is followed by a slower intramolecular lattice expansion in the 100 ns to 100 µs region. This second contribution to intramolecular expansion (and associated molecular volume change) is hypothesized as due to the thermal lattice expansion resulting from the energy of the laser pulse that increases the local temperature [51]. In addition, the penetration depth of the laser is finite, and this can cause local temperature gradients. The homogenization of the temperature across the crystal takes time and appears to occur within 10–100 µs [51]. This was confirmed by measuring the evolution of the isotropic Debye-Waller temperature factor ΔB with time (Figure 7c), as an indicator of the atomic thermal motion [51].

**Figure 8:**
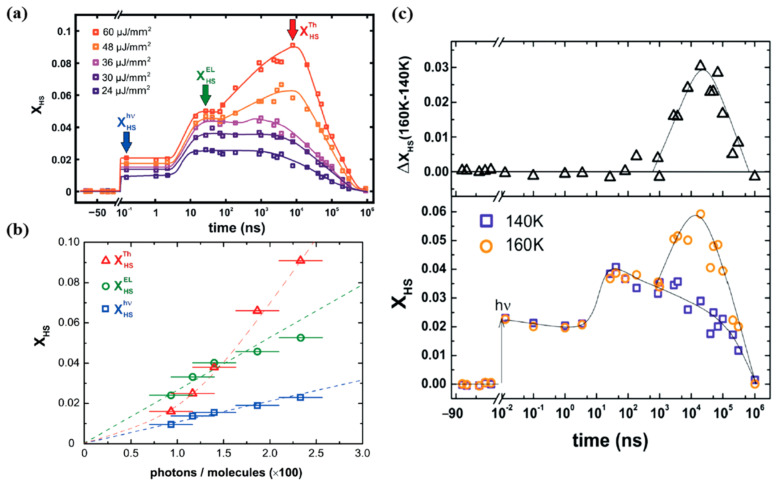
(**a**) The femtosecond laser excitation of low spin [Fe(Phen)_2_(NCS)_2_] to the high spin state at 140 K for different excitation energy densities. The photoinduced, elastic, and thermal steps to the high spin state are denoted as “hν”, “El”, and “Th” respectively. (**b**) The fraction of high spin state occupancy, X_HS_, with respect to the number of incident photons per 100 molecules. (**c**) The bottom panel shows the comparison of the photo-response, for the same excitation energy density of 30 µJ mm^−2^ at 140 K and 160 K. The top panel shows the difference between the high spin fraction between 140 K and 160 K. Reproduced with permission from Ref. [66]. Copyright 2016 The Royal Society of Chemistry.

A study performed by Betorni and coworkers [66], for different incident photon densities, on [Fe(phen)_2_(NCS)_2_] orthorhombic crystals, confirms the three steps model for conversion to the HS state at 140 K, as illustrated in Figure 8a [66]. Both the photoinduced and elastic steps appear for all excitation energy densities employed, while the thermal step only appeared for excitation energy densities exceeding a threshold of 40 µJ mm^−2^, in the 10 µs time scale.

The HS state occupancy fraction of the [Fe(phen)_2_(NCS)_2_] crystal, resulting from photo-excitation, elastic and thermal generation, is shown in Figure 8b with respect to the excitation energy densities at 140 K [66]. The photo-switching leads to a HS state fraction that linearly depends on the excitation energy density, as expected. Each photon appears to switch one [Fe(phen)_2_(NCS)_2_] molecule to the high spin state [66]. This efficiency of the direct photo-transition to the high spin state is close to unity, for SCO [Fe(phen)_2_(NCS)_2_] solids. This is similar to the result found in SCO molecular liquids [77,79]. The elastic step contribution leading to a transition to the high spin state also shows a linear relation with the incident photons but the response is 2.5–3 times larger than the photoswitching [66]. The relation between the HS state fraction and the incidents photons, for the elastic step in Figure 6a, indicates that one photon promotes approximately 2.66 molecules to the HS state (Figure 8b). The photo-response of [Fe(phen)_2_(NCS)_2_] then results in a thermal step that shows a non-linear relation with the excitation energy density [66]. Figure 8c shows the high spin fraction with a constant excitation energy density of 30 µJ mm^−2^ at two different temperatures 140 K and 160 K. The thermal step appears only at 160 K in the µs time delay region [66]. There is a volume expansion between 140 K and 160 K at the same excitation energy density for time delays in the µs region that indicates a laser induced heating of about 20 K. The system is then at the onset of the thermal spin crossover transition temperature (180 K for this crystal) when the sample temperature is set at 160 K. An excitation energy density of 30 µJ mm^−2^ is therefore large enough to induce a thermal transition at 160 K, while being ineffective at 140 K. This type of power and sample temperature behavior was also seen for [Fe^III^(3-MeO-SalEen)_2_]PF_6_ crystals [59], as discussed below. Thermally induced HS state population can therefore be avoided by using low-enough excitation energy density or by performing experiments at low-enough temperatures far away from the transition temperatures [66].

The limited fraction in the HS state at short time scales and the long time scales for much higher fractions transitioning to the HS state is a concern for device integration considerations [8]. This may be addressed by size scaling as discussed next

## 5. How Size Matters for Switching Dynamics

The capability to fabricate SCO materials in a small ensemble of molecules (nanoparticles) has opened the way to use the SCO phenomena in more applications as this may mean faster “write” speeds as discussed below. This could be important to many applications [8], and we suggest that faster write speeds may be accessible. In the previous section, we summarized the current understanding of the spin state switching dynamics in solids, where the equilibrium photo-induced HS state can proceed through three processes (photo, elastic, and thermal switching) with various time scales (ps, ns, and μs, respectively).

In the first approximation, the elastic propagation through an object of length scale *L* should happen within a time

(1) $$\tau_{el}\approx v_{ph}L.$$

The size-independent phonon speed $v_{ph}$ is tied to the speed of sound [80]. $\tau_{el}$ can therefore reach the ns time scale when the size of the material drops below the µm.

The thermal relaxation can be related to the thermal diffusivity coefficient $D_{T}$

(2) $$\tau_{T}\approx \frac{L^{2}}{D_{T}}.$$

For a diffusion coefficient *D_T_* = 2.6 × 10^−7^ m^2^ s^−1^ [85] if *L*= 1 µm, then *τ_T_* = 40 ns.

**Figure 9:**
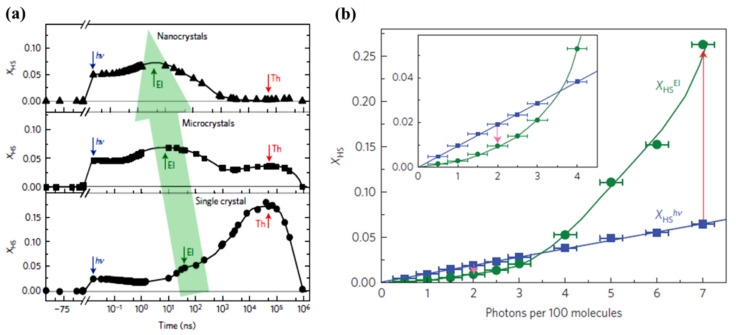
(**a**) The response of different sizes of low spin Fe^III^(3-MeO-SalEen)_2_]PF_6_ crystals to a femtosecond laser excitation, top panel: nanocrystals, middle panel: microcrystals, and bottom panel: single crystals. The photoinduced step, the elastic step, and the thermal step are denoted as, hν, El, and Th respectively. The green arrow indicates the elastic step shift towards the shorter time scale with the crystal size reduction. (**b**) The high spin fraction XHS formation due to photoinduced (measured at 10 ps) and elastic step (measured at 100 ns) with laser excitation energy density scaled in photons per 100 molecules. Reprinted with permission from Ref. [59]. Copyright 2016 Springer Nature.

Dynamic studies performed on the same molecular SCO system, but with different sizes, [59] showed experimentally that reducing the size of the crystal can impact the time scale of the elastic step associated with the spin state change in the ensemble (Figure 9a). The fraction of HS state molecules created by photo-generation increases when the size of the system decreases down to a size below the light penetration depth. In contrast, the authors found a HS state fraction triggered by thermal switching that increases with the size of the crystal. This increasing high spin state occupancy is related to a heat relaxation time roughly independent of the sample size, in contrast to Equation (2), also previously observed by the same authors [86]. This shift of more molecules to the HS state is possibly due to a high concentration of the SCO material and the decreased surface/volume ratio limiting the heat diffusion away from the SCO material [59]. Contrasting with this photo-thermal effect, the elastic step (the switching step that occurs from the elastic stress that generates from the internal pressure due to lattice expansion) shifts towards a shorter time scale by two orders of magnitude under size reduction. This photo-elastic effect phenomenon follows the expectation of Equation (1) and the delay time reaches the ns scale when the size of the material is a few hundred nm. Note, however, that the expected time scale of Equation (1) is shorter when estimated using the speed of sound, than the delay time found in experiments [59]. This suggests that the photo-elastic driven spin state transition has an origin more complex than a simple single step lattice expansion.

**Figure 10:**
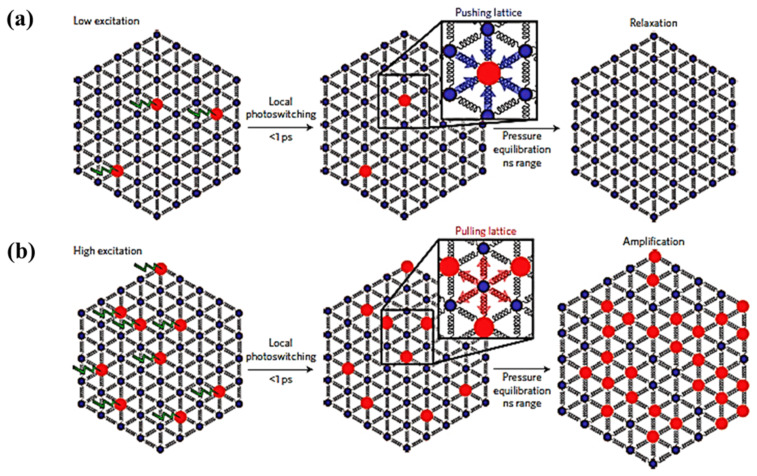
The schematic representation of the light induced cooperative phenomena, (**a**) At low excitation energy density only few molecules photo-switch to the high spin state and they can then relax back to low spin state under the pressure of low spin lattice, (**b**) At high excitation energy density more molecules will be photo switched to high spin which expands the volume of the lattice and it will pull more low spin molecules to high spin. Reprinted with permission from Ref. [59]. Copyright 2016 Springer Nature.

The elastic contributions resulting from LS to HS state switching can possibly be enhanced by exploiting intermolecular cooperativity to improve the photo switching efficiency of the LS to HS state transition [59]. The fraction of molecules photo-switched to the HS state linearly depends on the energy density used and is limited to one molecule per photon (as noted above), whereas the elastic step is shown to exhibit a nonlinear relation with laser power (Figure 9b). The nonlinear response indicates that there is a threshold excitation energy density where there is a HS fraction increment triggered by a amplification process [59]. The cooperative mechanism by which this light induced phenomena acts through nearest neighbors is schematically illustrated in Figure 10 [59]. When the excitation energy density is low (Figure 10a), the fraction of photo-switched HS molecules is limited to a linear response dependence. The molecules to be photo-switched to the HS state need a larger volume within a lattice. For a lattice with most molecules in the LS state, a molecule in the HS state is subjected to local elastic forces pushing HS spin state complex back to the previous LS equilibrium state. When the excitation energy density is large enough (Figure 10b) photo-induced HS state fraction increases sufficiently and exerts pressure on the lattice causing a lattice expansion [59]. This expansion favors the HS phase which then grows from nucleation sites and in turn increases the HS state fraction further. This self-amplification process, due to intermolecular cooperative phenomenon, can therefore enhance the photo switching efficiency, with reported values of more than 5 molecules per incident photon [59].

**Figure 11:**
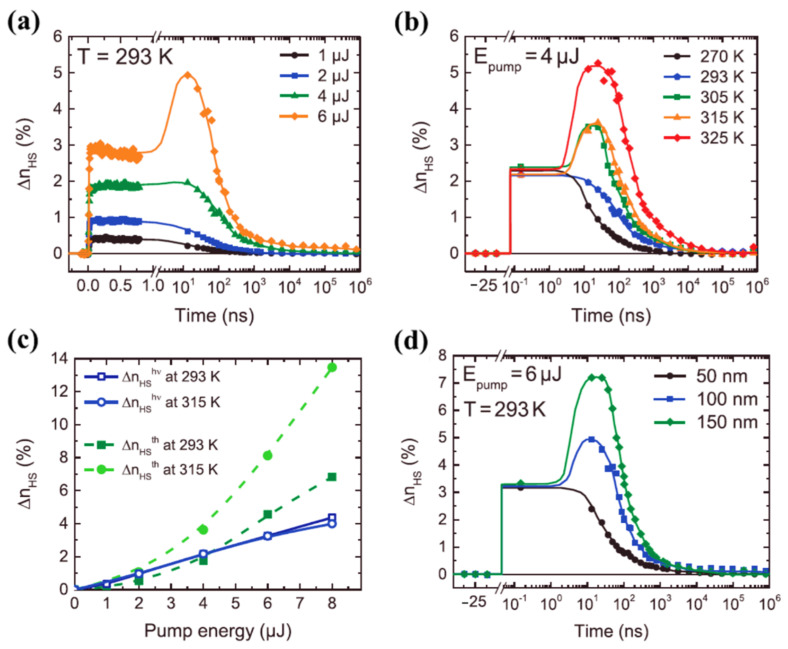
(**a**) The high spin state molecular fraction of a 100 nm [Fe(HB(tz)_3_)_2_] thin film with respect to the time delay, for different excitation energies. (**b**) At constant excitation energy, the time evolution of the high spin fraction of 100 nm thick thin film for different temperatures. (**c**) The comparison of high spin fraction formed due to photo-switching and thermal step at two different temperatures with respect to the pump energy. (**d**) The photo-response of a thin film of [Fe(HB(tz)_3_)_2_] to a excitation energy of 6 µJ, although of a different film thickness. Reprinted with permission from Ref. [87]. Copyright 2019 John Wiley and Sons.

While the thermal mediated optical switching temperature dependence of very different spin crossover molecules can be very similar, size plays a significant role, as noted above. The effect of size scaling was further extended to spin crossover molecular thin films, made of vacuum-evaporated [Fe(HB(tz)_3_)_2_] (tz = 1,2,4-triazol-1-yl) SCO complex, by Ridier et al. [87], as seen in Figure 11. The photo-response of 100 nm thick [Fe(HB(tz)_3_)_2_] SCO molecular film at room temperature for different excitation energies on the ns to ms time range is shown in Figure 11a [87]. As observed in single crystals and nano crystals, the HS fraction increases with increasing excitation energies (4 µJ and 6 µJ) (Figure 11a) through a two-step response, where the second response was observed in the 20–40 ns time window. [Fe(HB(tz)_3_)_2_] (tz = 1,2,4-triazol-1-yl) thin films [87] and [Fe(Phen)_2_(NCS)_2_] crystals [66] exhibit similar optical switching responses (note the similarity 8a and 11a in their laser power dependence and the similarity of 11b and 8c in their temperature dependence). The thermal switching step to the HS state has an efficiency increasing with temperature, when approaching the transition temperature of the [Fe(HB(tz)_3_)_2_] thin films [87], as in Figure 11b. Like the molecular [Fe(phen)_2_(NCS)_2_] SCO system [66], at temperatures near the SCO transition temperature, photo-thermal effects driving the system to the HS states are effective for [Fe(HB(tz)_3_)_2_] thin films (Figure 11b) but there was no direct evidence of an elastic relaxation peak [87]. Figure 11c shows the HS state fraction due to both photoinduced and thermal steps with different excitation energies, measured at 150 ps and 25 ns respectively, at two different temperatures 293 K and 315 K [87], and Figure 11c confirms the thermal origin of the conversion to the high spin state between 1 to 1000 ns. The observed two-step response was interpreted as a combination of direct switching with a secondary thermal switching due to laser-induced heating [87]. The second HS state population step, at higher excitation energies, is absent when the [Fe(HB(tz)_3_)_2_] film thickness is reduced (Figure 11d) [87]. This missing secondary thermal switching to the HS state of [Fe(HB(tz)_3_)_2_] could be the result of enhanced thermal dissipation through the substrate that does not allow the thermal transition to take place in [Fe(HB(tz)_3_)_2_] thin films. The thermal induced HS fraction shows a nonlinear behavior and a strong increase in the HS state fraction at higher temperatures. In the switching of [Fe(HB(tz)_3_)_2_] thin films, using a constant excitation energy, the photoinduced HS fraction increases linearly with the excitation energies and independently of the temperature. It has been shown that the intersystem crossing in the switching process takes about 171 fs for the 50 nm thin film and the vibrational cooling occurs within 2.4 ps [87].

Again, there is also ‘back’ conversion from the HS state obtained via optical excitation to the LS state for SCO systems. As the temperature approaches the thermal transition, [Fe(HB(tz)_3_)_2_] thin films relax back to the LS state, which occurs on a short time scale, down to 100 ns for the 50 nm thin film [87]. This introduces the next section, detailing the studies of the recovery dynamics of the initial LS state from the photoexcited HS metastable state.

## 6. Relaxation of the Transient Light-Induced State

**Figure 12:**
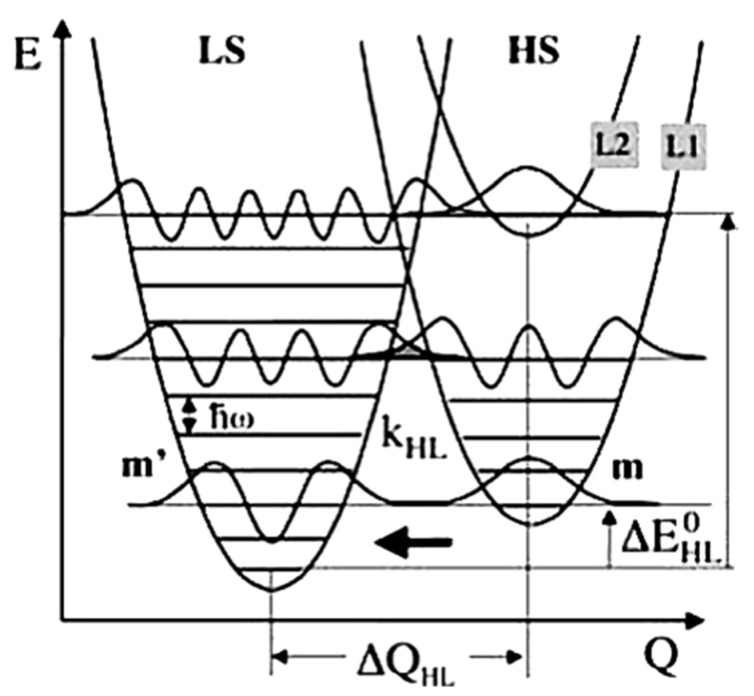
The high spin and low spin state potential wells with respect to the reaction coordinate. At low temperatures effective quantum tunneling occurs from the lowest vibrational level of the high spin state to the vibrational levels in the low spin state. Reprinted with permission from Ref. [72]. Copyright 1969 Springer Nature.

Studies done on the relaxation of SCO molecular materials from the HS to LS states, before the discovery of LIESST, were performed at ambient temperature, on materials with transition temperatures around ambient. The relaxation rate constant k_HL_ found for these materials was around 10^6^–10^8^ s^−1^ [70]. In 1980, Buhks et al. [88] theoretically described the relaxation process from the HS to LS states as a non-adiabatic multiphoton process which occurs via tunneling at low temperatures. In 1987, Xie and Hendrickson [89] showed experimental evidence for the low temperature tunneling process for a spin crossover complex embedded in a polymer matrix. The ligand sphere of the SCO complexes, lattice effect, and the external pressure due to the large difference in the metal-ligand bond length and the molecular volume can affect the HS to LS relaxation rate. The HS and LS state potential wells are shown in Figure 12. The relaxation rate via the tunneling process is given by Equation (3) [72,75].

(3) $$k_{HL}=\frac{2\pi }{\hbar^{2}\omega }\beta_{HL}^{2}F_{p}\left(T\right)$$

Here *β_HL_* is the electronic coupling matrix element given by the second order spin-orbit coupling and *ℏω* is the vibrational energy. *F_p_*(*T*) is the thermally averaged Franck-Condon factor given by Equation (4) [72,75].

(4) $$F_{n}\left(T\right)=\frac{\sum_{m}{\left|\langle X_{m+p}|X_{m}\rangle \right|}^{2}e^{\frac{-m\hbar \omega }{k_{B}T}}}{\sum_{m}e^{\frac{-m\hbar \omega }{k_{B}T}}}$$

$\langle X_{m+p}|X_{m}\rangle$ (*m* + *p* = *m*’) is the tunneling probability for a non-radiative process from a vibrational level m in the high spin to a vibrational level *m*’ of the low spin state (as schematically indicated in Figure 12) [72,75]. Here *p* is the reduced energy gap, a dimensionless measure of the vertical displacement of the potential wells, equal to Δ$E_{HL}^{0}$/*ℏω*. The vertical displacement of the potential wells Δ$E_{HL}^{0}$ is the zero-point energy difference between the LS and HS states. At low temperatures tunneling occurs from the lowest vibrational level of the high spin state to the low spin state and the relaxation constant is given by Equation (5) [72,75].

(5) $$k_{HL}\left(T\to 0\right)=\frac{2\pi }{\hbar^{2}\omega }\beta_{HL}^{2}{\left|\langle X_{n}|X_{0}\rangle \right|}^{2}$$

**Figure 13:**
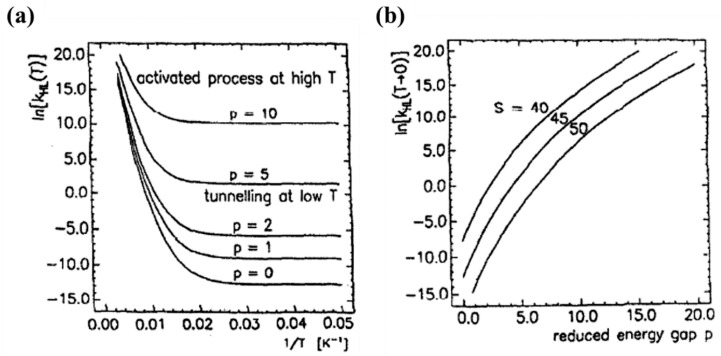
(**a**) The relaxation rate constant *k_HL_* in logarithmic scale as a function of 1/*T* at various reduced energy gaps *p*, with S = 45, *ℏω* = 250 cm^−1^ and *β_HL_* = 150 cm^−1^. (**b**) The relaxation rate at *T* → 0 with respect to the reduced energy gap *p* at various S values. Reprinted with permission from Ref. [75]. Copyright 1991 Elsevier.

For low temperatures, the Franck-Condon *F_p_*(*T*) factor is given by ${\left|\langle X_{n}|X_{0}\rangle \right|}^{2}=\frac{S^{p}e^{-S}}{p!}$, where S is the Huang–Rhys factor S = ½f $\Delta Q_{HL}^{2}$/*ℏω* with Δ*Q_HL_* = √6Δr_HL_. S is a dimensionless measure of the horizontal displacement of the potential wells [72,75]. For Fe (II) spin crossover compounds, the value of *β*_HL_ is around 150 cm^−1^ and Δ*Q_HL_* = 0.5 Å. The value of the Huang-Rhys factor, which is about 45, can be estimated using a vibration energy (*ℏω*) of 250 cm^−1^ and an average force constant of 2 × 10^5^ dyn/cm. The relaxation rates on logarithm scale with respect to 1/*T* for different dimensionless reduced energy gap *p* values are shown in Figure 13a [75]. At low temperatures, the relaxation rate is temperature independent, which indicates a tunneling process from the lowest vibrational level of the high-spin state through the barrier to vibrational levels in the low-spin state. The relaxation rate increases exponentially with increasing the *p* value at low temperatures [75]. At low temperatures the relaxation rate increases when increasing the energy gap which separates the ground state energies of the HS and LS states. The SCO transition temperature is a measure of the zero-point energy gap and increases with the transition temperature. This ‘inverse-energy-gap-law’ assumes that the metastable LIESST state has a lifetime inversely proportional to the SCO transition temperature *T*_1/2_ [75]. The relaxation rate plotted with respect to the dimensionless reduced energy gap *p* is shown in Figure 13b and the relaxation rate at low temperatures decreases with increasing S value. The relaxation rate is also governed by the metal ligand bond length [75].

Relaxation kinetics studies have been investigated for several SCO complexes using time resolved spectroscopic techniques. For [Fe(bipy)_3_]^2+^ in aqueous solution, the relaxation time back to the more stable LS states is approximately 0.6 ns [75] and for [Fe(tren(py)_3_)]^2+^ in acetonitrile is 60 ns [90]. The lifetime of the HS of [Fe(phen)_3_](BF_4_)_2_ (phen = 1,10-phenantroline) complex dissolved in acetonitrile is ~1.1 ns [91]. The studies on relaxation kinetics in solution are mostly conducted at room temperature in order to prevent solvent freezing. Therefore, the quantum tunneling effect has not been observed for molecules in the solution [39], but there are clearly solvent effects.

Reverse LIESST can be used to switch the HS state back to LS state [70,92], and one approach is to use near IR radiation to trigger the recovery of the initial and more stable LS state. Marino et al. studied the reverse LIESST pathway of [Fe(ptz)_6_](BF_4_)_2_ using ultrafast transient absorption spectroscopy [92]. With 830 nm irradiation, the [Fe(ptz)_6_](BF_4_)_2_ HS state is excited to the ^5^E state, a metal center weighted state, which permits an optically allowed spin state transition to occur. Immediately after irradiation, the ^5^E state relaxes to the ligand field state ^3^T_1_ via an intersystem crossing, with a time constant of 1.7 ps [92,93]. Next, the system reaches the LS state by undergoing another intersystem state crossing with a measured time constant of 39 ps [92]. Switching from the HS to LS state by means of reverse LIESST is evidently much faster than the relaxation time observed with tunneling. However, the quantum efficiency of the reverse LIESST is thought to be low and decreases with increasing temperature. Hence the resulting light induced spin state population is rather low [92].

For solid SCO solid molecular materials, the relaxation time has, so far, been seen to be typically in the 100 µs to 1 ms range [50,94]. Cooperativity also plays an important role in the relaxation of the HS state [72,95], as it does with other electronic state changes in spin crossover complexes, as already much emphasized. Due to the cooperative effects there will be an internal pressure created in the lattice matrix which speeds up the relaxation process [72,95]. This self-acceleration occurs as the energy barrier between the HS and LS reduces due to internal pressure. In materials without strong cooperativity, such as [Fe_x_M_1-x_(ptz)_6_](BF_4_)_2_ (with M = Zn, Mn, Cd, Ni and Co), in which the Fe(II)-SCO is diluted with non-SCO ion, result in relaxation rates due to the quantum tunneling, which then depends on the non-SCO ion in the low temperature region [72,75], while the thermally activated relaxation rate at high temperatures is not sensitive to the non-SCO ion. Internal pressure can also be seen in these non-cooperative materials. The bigger the non-SCO ion, the larger the internal pressure, hence the longer the lifetime of the HS state [39,72,75].

This opens the issue of very long-time stabilization of the light-induced HS state, which could be essential for applications. This type of condition is discussed in the next section.

## 7. Stabilization of the Light-Induced State

The needed experimental conditions for repetitive experiments in the previous sections implied that there is a constrained choice of experimental conditions (materials, temperatures) where the relaxation of the HS time was long enough to avoid perturbing the LS → HS studies and short enough to allow appropriate repetition rates. For applications, in particular targeting memory devices, not only is a ‘write’ mechanism required, but stabilization of the HS states for very large time scales is important—memory requires at least some nonvolatility. While LIESST is a possible approach for stabilizing the light-triggered HS state, a long-lasting metastable HS state requires low transition temperature. Low temperatures much smaller than the thermal *T*_1/2_ spin state transition temperature are needed to preserve the metastable HS, so operations are, therefore, limited to temperatures not exceeding a few tens of K at best.

It is well known that the spin state transition exhibits temperature hysteresis as the result of cooperativity effects [96], opening the door to isothermal bistability. This bistability has been studied for different spin crossover systems using pulsed laser or continuous laser irradiation [97,98,99,100,101,102] at temperatures within the spin state transition thermal hysteresis loop. Once the molecular spin state has switched to the HS state, the retention of the HS state can be very long, as the system is at temperatures where bistability is possible. Furthermore, one can also take advantage of thermal effects to trigger or increase the HS population, as detailed in the previous section. If the initial temperature is within the thermal hysteresis loop, and one exceeds the LS → HS transition temperatures during photo-irradiation, then the HS fraction is stabilized when thermal equilibrium is reinstated [103]. A typical experimental signature of such effect is the influence of laser fluence, where larger temperature increases occur when increasing fluence [101]. Furthermore, increased heating also speeds up the switching time, down the 10 ns time range for the highest fluence values, on nanoparticles [99]. This behavior is confirmed by time-resolved electron microscopy, showing a sub-µm particle expansion under illumination, that fully takes place below 20 ns for the highest fluence used [104,105]. Generally, one should be cautious about the thermal heating processes, as SCO materials may have limited thermal conductivity [106].

In short, for device applications, the targeted switching time scale should be below 1 ns, which is a rather challenging time scale for SCO systems made of an ensemble of molecules in interactions. To achieve a competitive device, via permanent photo-writing operation, size scaling to small dimensions is going to be advantageous.

## 8. Conclusions

Spin crossover molecular materials have gained considerable attention since their discovery in the 1920’s [107,108,109,110,111,112]. The discovery of the LIESST effect [70,71,72] has opened the door to device applications where light can be used to trap both spin states at low temperatures for a significantly long time, but has also made possible further investigations of the optically driven spin state switching processes. Photo-thermal switching can further extend the operational temperature for molecular spin state switching, within a range defined by the hysteresis in the thermal transition curves of the SCO material. While the spanning in temperature remains limited, nevertheless, it can, however, reach more ambient temperatures for appropriate families of SCO materials, therefore making several SCO complexes possibly more relevant for applications. Cooperativity is here a key necessary property: it allows higher temperatures operation, as well as propagation and amplification of the statistics of the light-induced switching at the molecular level.

Determining the time scale for the photo-switching of SCO materials is a very rich and complicated problem, owing to the sensitivity of the SCO molecules to their environment and the multiple types of stimuli capable to trigger or impede the spin transition. For independent molecules, like molecules in solutions, the consensus in the literature is that the photoinduced switching (from the stable to the metastable states) occurs in a time scale in the ps range [66,77,79,81,82,83]. For molecules with intermolecular interactions, like those in solid materials, cooperativity makes the elastic and thermal propagation times dominate the switching speed, with typical reported state switching times in the ns and µs time scales respectively [51,63,66]. These times however depend on the size of the material (or the irradiation spot), roughly linearly increasing with the length scale, driven by the propagation of acoustic waves and diffusion of heat respectively.

For applications near ambient temperatures, optical switching requires a thermal heating process. Here, there is a trade-off between high light fluences for shortening the switching time, and sample resilience to multiple switching events. The switching endurance is a key application issue, as fidelity and reliability are often a concern for molecular devices. A broad understanding of the limits to fidelity and reliability is, however, not extensive given the limited number of studies in the literature (see a pertinent review in [113]). Even though there are indications that laser pulses of high fluence allow for only a limited number of operations [105], recent reports indicate that SCO materials should be capable of sustaining a significant number of thermal switching events (up to 10^7^) [114]. Further improvements can also be gained by nanomaterials design. One example is an experimental evidence that the required laser power for photo-switching the spin state can be reduced by combining SCO particles, coated with silica, with gold nanoparticles [115]. Exciting the plasmon resonance of the gold nanoparticles, leads to the Au nanoparticles acting as local nano heaters [115,116], with indications of lower fluence needed for switching [117] and faster thermal switching related to a more efficient heating process [105]. With the help of better material design, in the perspective of using smaller SCO particles, one can consider that the optical device applicability remains an open issue.

Spin crossover materials on the nanoscale are clearly the favored route for reaching the shortest switching times while keeping the advantage of cooperativity, as both are essential to functioning devices that can compete with CMOS. We, therefore, identify nanoparticles or patterned areas in thin films as ideal candidates for devices incorporating SCO for fast and reproducible light-triggered switching. As a rule of thumb, the spin state switching time can be reduced to the ns if the SCO molecular cluster size is reduced to 10 nm. Sub-ns operation is expected to require SCO materials at the dimensions of a few nm only. The key issue is however to better understand and control the SCO material cooperativity when there is a reduction in spatial dimensions. Clearly, the effect of size is a long-standing issue in the study of SCO properties. For example, reducing the size of nanoparticles from the 100 nm to the 10 nm scale weakens cooperativity, which surprisingly reappears with further reduction down a few nm [118]. This is most probably related to surface effects [119], suggesting that the environment of the SCO becomes of critical importance for such nm size range. Several open questions and possible experimental routes for fast SCO switching are therefore envisioned, beyond the need to reduce the active material size down the nm range. Of particular interest is the possibility to use the electric dipolar environment of the SCO to influence the stability, and possibly the cooperativity of the SCO states, for example by means of an adjacent ferroelectric, or ferroelectric co-crystal environment as the best asymptotic case [37].

All the spin dynamics and switching speeds that have been discussed here are about spin state switching using optical excitation. If the spin transition is triggered by an electric field [120,121,122,123,124,125,126,127,128,129] or by switching of the dipoles of the environment [8,42,130,131], fast electronic-driven switching could occur, within temperature range related to the thermal transition temperatures of the material. In these cases, however, the experimentalist does not have the advantage of fast pump-probe optics to get insight into the dynamics. The main experimental bottleneck to the study of voltage driven spin state switching is the low-conductivity (even insulating) and high impedance of most SCO complexes and hence of the related molecular devices [8]. Indirect measurements using graphene electrical detectors, shown for LIESST [132] and thermal transition [133] of SCO thin films or nanoparticles, can possibly take advantage of the RF graphene electronics circuit. These hybrid devices open the door to probing electronic properties of SCO devices down the ns time range, as well as making possible high-frequency operation of such switching organic devices.

Time-resolved electronics studies are therefore timely to study how fast the material switching and dipole switching will occur, owing to the technological importance of the writing process in a memory cell. Recent studies have been performed on molecular multiferroic spin transistors, where the ferroelectric layer affects the spin state [130,131]. Therefore, the switching speed can also be governed by the ferroelectric material, not the SCO molecular system, in these particular devices. If the ferroelectric materials have the ability to implement fast polarization reversal, at modest voltages, then accelerating the switching speed of the SCO molecules might become the rate limiting step.

In summary, we argue here that SCO materials of a few nm in size are ideally suited for fast optical device switching between spin states and thus obtaining more competitive writing speeds and spin state operation processes. For voltage controlled switching there is presently less known about the limits to the write speed. Room temperature operation, resistance to fatigability, electrical excitation, and detection schemes are key future directions for further studies, necessary to assess the viability of SCO materials for device operations. In spite of the unknowns, the multiplicity of approaches for synthesis and control of the material environment and the initial results on test systems [130,131] and hybrid circuits [8] are encouraging, making SCO complexes a potentially unique organic material for optoelectronics.

## Data Availability

Full data and analysis performed by the authors is available on request.
